# *nab*-Paclitaxel–Based Therapy in Underserved Patient Populations: The ABOUND.PS2 Study in Patients With NSCLC and a Performance Status of 2

**DOI:** 10.3389/fonc.2018.00253

**Published:** 2018-07-24

**Authors:** Ajeet Gajra, Nagla Abdel Karim, Deborah A. Mulford, Liza Cosca Villaruz, Marc Ryan Matrana, Haythem Y. Ali, Edgardo S. Santos, Tymara Berry, Teng Jin Ong, Alexandra Sanford, Katayoun Amiri, David R. Spigel

**Affiliations:** ^1^Oncology, ICON Research, North Wales, PA, United States; ^2^Division of Hematology Oncology, University of Cincinnati, Cincinnati, OH, United States; ^3^Department of Medicine, Hematology/Oncology, University of Rochester Medical Center, James P. Wilmot Cancer Center, Rochester, NY, United States; ^4^Department of Internal Medicine, Hematology/Oncology, University of Pittsburgh, Pittsburgh, PA, United States; ^5^Department of Internal Medicine, Hematology/Oncology, Ochsner Medical Center, New Orleans, LA, United States; ^6^Department of Internal Medicine, Medical Oncology, Henry Ford Health System, Detroit, MI, United States; ^7^Hematology/Oncology, Eugene M. and Christine E. Lynn Cancer Institute, Boca Raton, FL, United States; ^8^Drug Safety, Celgene Corporation, Summit, NJ, United States; ^9^Medical Affairs, Celgene Corporation, Summit, NJ, United States; ^10^Biostatistics, Celgene Corporation, Summit, NJ, United States; ^11^Department of Internal Medicine, Medical Oncology, Sarah Cannon Research Institute, Nashville, TN, United States

**Keywords:** chemotherapy, maintenance therapy, *nab*-paclitaxel, non-small cell lung cancer, poor performance status

## Abstract

**Introduction:**

The phase II ABOUND.PS2 study (NCT02289456) assessed safety/tolerability of a first-line modified *nab*-paclitaxel/carboplatin regimen for patients with advanced non-small cell lung cancer (NSCLC) and Eastern Cooperative Oncology Group (ECOG) performance status (PS) 2.

**Methods:**

Chemotherapy-naive patients with stage IIIB/IV NSCLC and ECOG PS 2 received four cycles of *nab*-paclitaxel 100 mg/m^2^ days 1 and 8 plus carboplatin area under the curve 5 day 1 q3w (induction). Patients without progression received *nab*-paclitaxel monotherapy (100 mg/m^2^ days 1 and 8 q3w) until progression/unacceptable toxicity. Primary endpoint: percentage of patients discontinuing induction due to treatment-emergent adverse events (TEAEs).

**Results:**

11/40 treated patients (27.5%; 95% CI, 14.60–43.89) discontinued chemotherapy induction due to TEAEs; 16/40 (40.0%) continued *nab*-paclitaxel monotherapy. Median progression-free and overall survival were 4.4 (95% CI, 2.99–7.00) and 7.7 (95% CI, 4.93–13.17) months. Grade 3/4 TEAEs during induction included neutropenia (22.5%), anemia (17.5%), thrombocytopenia (5.0%), and peripheral neuropathy (2.5%).

**Conclusion:**

This *nab*-paclitaxel–based regimen was tolerable in patients with advanced NSCLC and ECOG PS 2, with efficacy comparable to historical chemotherapy data.

## Introduction

Performance status (PS) has been shown to strongly predict survival in patients with non-small cell lung cancer (NSCLC) and, therefore, is an important consideration when selecting treatment options (1). Although patients with poor PS [Eastern Cooperative Oncology Group (ECOG) PS ≥ 2] account for 30–50% of those with advanced lung cancer in clinics, they are typically underrepresented in clinical trials possibly due to concerns about tolerability associated with experimental treatment, deterioration of PS, and reduced efficacy relative to patients with a good PS (2–6). In patients with advanced NSCLC and ECOG PS 2, overall survival (OS) has been demonstrated to be worse than in those with ECOG PS 0 or 1 (7, 8).

Chemotherapy with both platinum doublets (non-cisplatin-based) and single agents is recommended as first-line treatment of patients with advanced NSCLC and ECOG PS 2 (9). Treatment with single agents has been associated with an improved toxicity profile relative to combination regimens (10). Although many patients are treated with single agents, subset analyses from several historical studies, including ECOG 1594 and CALGB 9730, have demonstrated the benefit of platinum doublets (7, 9, 11). Although some recent prospective trials of first-line platinum-doublet combination regimens have confirmed these findings, improved therapeutic options remain an unmet need given the concerns about toxicity with chemotherapy regimens in this patient population (3, 10, 12–16).

In a phase III trial, first-line *nab*-paclitaxel (100 mg/m^2^ days 1, 8, and 15 q3w) plus carboplatin [area under the curve (AUC) 6 q3w] significantly improved the primary endpoint [overall response rate (ORR) 33 vs 25%; *P* = 0.005] vs paclitaxel plus carboplatin in patients with advanced NSCLC and ECOG PS 0–1 (17). Significantly less grade ≥3 neuropathy, neutropenia, arthralgia, and myalgia but more thrombocytopenia and anemia were reported with *nab*-paclitaxel plus carboplatin vs paclitaxel plus carboplatin. In addition, a modified version of the phase III treatment regimen (*nab*-paclitaxel 100 mg/m^2^ days 1 and 8 plus carboplatin AUC 5 mg⋅min/mL, both q3w) administered as induction chemotherapy followed by radiotherapy combined with erlotinib reported a median progression-free survival (PFS) and OS of 10 and 13 months and an acceptable safety profile in a subset of patients with ECOG PS 2 enrolled in a phase II trial (*n* = 48/75) (18).

The phase II ABOUND.PS2 study was therefore designed to further characterize the safety, tolerability, and efficacy of a platinum-doublet regimen in patients with advanced NSCLC and ECOG PS 2 by using a modified *nab*-paclitaxel plus carboplatin regimen [approved dose of *nab*-paclitaxel (100 mg/m^2^) but modified schedule (days 1 and 8) with carboplatin AUC 5 on day 1], and also to examine the effects of continued maintenance therapy with *nab*-paclitaxel. Safety, efficacy, and quality-of-life (QOL) outcomes after four cycles of *nab*-paclitaxel plus carboplatin treatment followed by *nab*-paclitaxel monotherapy are reported.

## Materials and Methods

### Study Population

Patients with stage IIIB or IV, histologically or cytologically confirmed NSCLC measured by Response Evaluation Criteria in Solid Tumors (RECIST) version 1.1 who were not a candidate for curative surgery or radiation therapy were enrolled in this study. Key eligibility requirements included age ≥18 years; no prior anticancer therapy for the treatment of metastatic disease (adjuvant treatment permitted providing cytotoxic chemotherapy was completed 12 months before consent and without disease recurrence); ECOG PS 2; and adequate hematologic, renal, and liver function. Patients with active brain metastases or pre-existing peripheral neuropathy grade ≥2 [as per the National Cancer Institute’s Common Terminology Criteria for Adverse Events version 4.0 (CTCAE v4.0)] were excluded.

This study was conducted in accordance with the Declaration of Helsinki and Good Clinical Practice Guidelines of the International Conference on Harmonisation. Informed consent was obtained from all patients before study entry. The trial is registered at ClinicalTrials.gov (NCT02289456).

### Study Design

The phase II, single-arm, open-label, multicenter ABOUND.PS2 study was conducted at seven sites in the United States. Enrolled patients received *nab*-paclitaxel plus carboplatin for four cycles (induction), followed by monotherapy with *nab*-paclitaxel if eligible. During induction, patients were treated with *nab*-paclitaxel 100 mg/m^2^ intravenously on days 1 and 8 plus carboplatin AUC 5 mg⋅min/mL intravenously on day 1 every 21 days for four cycles. After completion of induction therapy and in the absence of disease progression, patients could continue in the study and receive monotherapy with *nab*-paclitaxel 100 mg/m^2^ intravenously on days 1 and 8 every 21 days until progression or unacceptable toxicity. A follow-up visit occurred 28 days after the last dose of study drug and, thereafter, approximately every 90 days for up to 1 year after the last patient was enrolled.

The primary endpoint (also the primary safety endpoint) was the percentage of patients who discontinued study treatment during induction due to treatment-emergent adverse events (TEAEs), defined as any adverse event or serious adverse event occurring or worsening on or after the first dose of study drug through 28 days after the last dose of the study drug. In addition, any serious adverse event with onset >28 days after the last dose of study drug that was assessed by the investigator as related to the study drug was considered a TEAE. Secondary endpoints included additional safety assessments, investigator-assessed PFS, disease control rate (DCR), OS, ORR, time to response (TTR), and duration of response (DOR)—all assessed during the entire study. PFS was defined as the time from the date of the first dose of study drug to the date of disease progression per RECIST v1.1 guidelines or death from any cause. DCR was defined as the percentage of patients who had continued stable disease or better. OS was defined as the time between the date of the first dose of study drug and death. ORR was defined as the percentage of patients who had a radiological complete or partial response during the course of the study per RECIST v1.1 guidelines. TTR was defined as the time from day 1 of study treatment to the first occurrence of complete or partial response; DOR was measured from the time that the measurement criteria were first met for complete or partial response (whichever was recorded first) until the first date that recurrent or progressive disease was radiologically documented. Exploratory endpoints included changes in QOL and lung function (spirometry parameters).

### Study Assessments

All patients who received ≥1 dose of study drug were included in the safety and efficacy populations. Throughout the study, safety was evaluated on days 1 and 8 of every cycle, at treatment discontinuation, and at follow-up. Adverse events were classified by the Medical Dictionary for Regulatory Activities, and severity was assessed according to CTCAE v4.0. Similar medical adverse event terms were grouped together to provide a more comprehensive presentation of safety. Tumor assessment *via* computed tomography scans occurred every two cycles (−3/+7 days) and continued until treatment discontinuation, withdrawal of consent, loss to follow-up, or death. The Lung Cancer Symptom Scale (LCSS) and the EuroQol 5 Dimensions 5 Levels (EQ-5D-5L) questionnaires were used to measure QOL. Patients answered each question using a visual analog scale (VAS) to indicate the symptom intensity. QOL was assessed on day 1 of cycles 1–4 throughout the study and at treatment discontinuation. ECOG PS was assessed by patients on day 1 of each cycle and at treatment discontinuation and by physicians at screening, on day 1 of each cycle, and at treatment discontinuation. Spirometry was used to assess patient lung function. Spirometry measurements, which included forced expiratory volume in 1 s (FEV_1_), forced vital capacity (FVC), and peak expiratory flow (PEF), were measured by physicians using a portable spirometer (approved by the US Food and Drug Administration) at screening, on day 1 of each cycle, and at treatment discontinuation.

### Statistical Analyses

The primary objective of this study was to assess the safety and tolerability of the combination regimen. The planned sample size of 50 patients was justified on the basis of the precision of the estimate of the primary endpoint. Safety and efficacy endpoint evaluations were based on the point estimates and the associated two-sided 95% CIs. Patients were considered to have met the criteria of the primary safety endpoint if they discontinued treatment before the start of the fifth cycle due to an adverse event. The percentage of patients meeting these criteria was calculated along with the corresponding exact binomial CI. Plots of Kaplan–Meier product-limit estimates were used to summarize the PFS and OS curves. The ORR was the percentage of all treated patients who achieved a best overall response of partial response or complete response; the DCR was the percentage of patients who attained any clinical response, including stable disease. Patients with available QOL data from baseline and ≥1 postbaseline visit were included in the QOL analyses. For statistical purposes, all scales were aligned so that a positive change from baseline indicated improvement. Changes from baseline LCSS and EQ-5D VAS-scaled items were described by descriptive statistics.

## Results

### Patients

Due to the slower-than-planned rate, enrollment was terminated early. A total of 40 patients were enrolled in this study from May 4, 2015, to July 31, 2016. The median age was 67.5 years, and 20.0% of patients were aged 70–74 years (Table 1). Most patients were white (92.5%), had nonsquamous histology (62.5%), and were male (60.0%).

**Table 1:** Baseline characteristics.

Patient characteristic	All treated patients (*N* = 40)
Age, median (range), years	67.5 (44–84)
65–69 years, %	12.5
70–74 years, %	20.0
≥75 years, %	27.5
Sex, *n* (%)	
Male	24 (60.0)
Female	16 (40.0)
Race, *n* (%)	
White	37 (92.5)
Black or African American	3 (7.5)
Histology, *n* (%)	
Nonsquamous	25 (62.5)
Squamous	15 (37.5)
Stage of disease at enrollment, *n* (%)	
IIIB	1 (2.5)
IV	39 (97.5)
Charlson comorbidity index score, *n* (%)	
0	8 (20.0)
1–2	24 (60.0)
3–4	8 (20.0)
Spirometry measurements	
FEV_1_, mean (SD), L	1.29 (0.475)
FVC, mean (SD), L	2.03 (0.676)
FEV_1_/FVC ratio, mean^a^ (SD)	0.65 (0.160)
PEF, mean (SD), L/s	2.66 (1.399)

*^a^n = 34 for FEV_1_/FVC ratio*.

*FEV_1_, forced expiratory volume in 1 s; FVC, forced vital capacity; PEF, peak expiratory flow*.

### Primary Endpoint

During induction, 24 patients (60.0%) discontinued treatment: 5 during/on completion of cycle 1, 4 during/on completion of cycle 2, 6 during/on completion of cycle 3, and 9 during/on completion of cycle 4. In total, 11 patients (27.5%; 95% CI, 14.60–43.89) discontinued due to TEAEs (primary endpoint; Table 2), 2 due to asthenia, and 1 each due to fatigue, pain, febrile neutropenia, neutropenia, drug hypersensitivity, lung abscess, dehydration, seizure, and dyspnea; no discontinuations were due to peripheral neuropathy.

**Table 2:** Primary endpoint (discontinuations due to TEAEs).

Parameter, *n* (%)	All treated patients (*N* = 40)
Patients who discontinued treatment during induction	24 (60.0)
During/upon completion of cycle 1	5 (12.5)
During/upon completion of cycle 2	4 (10.0)
During/upon completion of cycle 3	6 (15.0)
During/upon completion of cycle 4	9 (22.5)
Patients who discontinued during induction due to TEAE (primary endpoint)	11 (27.5)
Asthenia	2 (5)
Dehydration	1 (3)
Drug hypersensitivity	1 (3)
Dyspnea	1 (3)
Fatigue	1 (3)
Febrile neutropenia	1 (3)
Lung abscess	1 (3)
Neutropenia	1 (3)
Pain	1 (3)
Seizure	1 (3)
Patients who discontinued during induction due to	
Progressive disease	4 (10.0)
Symptomatic deterioration	4 (10.0)
Death^a^	2 (5.0)
Withdrawal by patient	2 (5.0)
Other	1 (2.5)
Patients who were treated in monotherapy	16

*TEAE, treatment-emergent adverse event*.

*^a^One death each due to malignant disease and unknown cause*.

### Treatment Exposure

During the entire study, all patients discontinued treatment (*N* = 40), primarily due to adverse events or progressive disease (12 patients each). During induction, 24 patients discontinued treatment; in addition to the 11 patients who discontinued due to TEAEs, 4 each did so due to symptomatic deterioration and progressive disease, 2 due to patient withdrawal, 2 due to death (malignant disease and unknown cause), and 1 due to other reasons (off study per physician decision). Of the 16 patients who discontinued treatment during monotherapy, the most common reason for discontinuation was progressive disease (8 patients), and only 1 patient discontinued due to a TEAE [generalized weakness (grade 2)].

In all patients receiving treatment during induction (*n* = 40), the median treatment duration was 2.76 months. The majority of patients (62.5%) completed induction therapy (four cycles of *nab*-paclitaxel plus carboplatin). The *nab*-paclitaxel median cumulative dose, dose intensity, and percentage of per-protocol dose during induction were 600.0 mg/m^2^, 55.08 mg/m^2^/week, and 82.62%, respectively. During induction, ≥1 *nab*-paclitaxel dose reduction, dose delay, and dose not administered occurred in 27.5, 42.5, and 50.0% of patients, respectively.

After completing induction, 16 patients were treated in the monotherapy part. The median treatment duration in these patients was 3.1 (range 0.7–10.4) months. The *nab*-paclitaxel median cumulative dose, dose intensity, and percentage of per-protocol dose during monotherapy were 700 mg/m^2^, 47.46 mg/m^2^/week, and 71.19%, respectively. During monotherapy, ≥1 *nab*-paclitaxel dose reduction, dose delay, and dose not administered occurred in 25.0, 50.0, and 25.0% of patients, respectively.

During the follow-up period, three patients received palliative radiotherapy but not concurrently with chemotherapy during the study. In total, 12 patients received a checkpoint inhibitor in a later line of therapy.

### Safety

Of the 40 patients treated during induction, 100.0% experienced a TEAE, 75.0% experienced a grade 3/4 TEAE (12.5% grade 4), and 50.0% experienced a serious TEAE. The most common TEAEs reported during induction included grade 1/2 nausea (52.5%; no grade 3/4), anemia (47.5%; 17.5% grade 3 only), fatigue (42.5%; 7.5% grade 3 only), neutropenia [35.0%; 22.5% grade 3/4 (Table 3); grade 3/4 febrile neutropenia accounted for 5.0%], asthenia (25.0%; 10.0% grade 3 only), and thrombocytopenia (20.0%; 5.0% grade 3 only); all-grade peripheral neuropathy occurred in 15.0% of patients (grade 3 occurred in one patient).

**Table 3:** Safety.

Treatment-emergent adverse events occurring in ≥20% of patients, *n* (%)	Induction part (*N* = 40)		Monotherapy part (*N* = 16)	
	All grade	Grade 3/4	All grade	Grade 3/4
**General myelosuppression**				
Anemia	19 (47.5)	7 (17.5)	5 (31.3)	1 (6.3)
Neutropenia	14 (35.0)	9 (22.5)	3 (18.8)	1 (6.3)
Thrombocytopenia	8 (20.0)	2 (5.0)	0	0
**Nonhematologic events**				
Nausea	21 (52.5)	0	1 (6.3)	0
Fatigue	17 (42.5)	3 (7.5)	5 (31.3)	0
Asthenia	10 (25.0)	4 (10.0)	2 (12.5)	1 (6.3)
Dehydration	10 (25.0)	3 (7.5)	1 (6.3)	0
Decreased appetite	10 (25.0)	2 (5.0)	0	0
Constipation	9 (22.5)	1 (2.5)	1 (6.3)	0
Alopecia	9 (22.5)	0	0	0
Diarrhea	8 (20.0)	1 (2.5)	1 (6.3)	0

Of the 16 patients who were treated with monotherapy, 87.5% experienced a TEAE, 43.8% experienced a grade 3 TEAE (no grade 4), and 25.0% experienced a serious TEAE. The most commonly reported TEAEs during monotherapy included anemia (31.3%; 6.3% grade 3), fatigue (31.3%; no grade 3), pneumonia (18.8%; 12.5% grade 3), neutropenia [18.8%; 6.3% grade 3 (Table 3); no febrile neutropenia], and asthenia (12.5%; 6.3% grade 3). No thrombocytopenia was reported during monotherapy. Pneumonia was the only grade 3 TEAE reported in >1 patient. No patients experienced grade 3 peripheral neuropathy during monotherapy, and one patient each experienced grades 1 and 2 peripheral neuropathy.

### Efficacy

**Figure 1:**
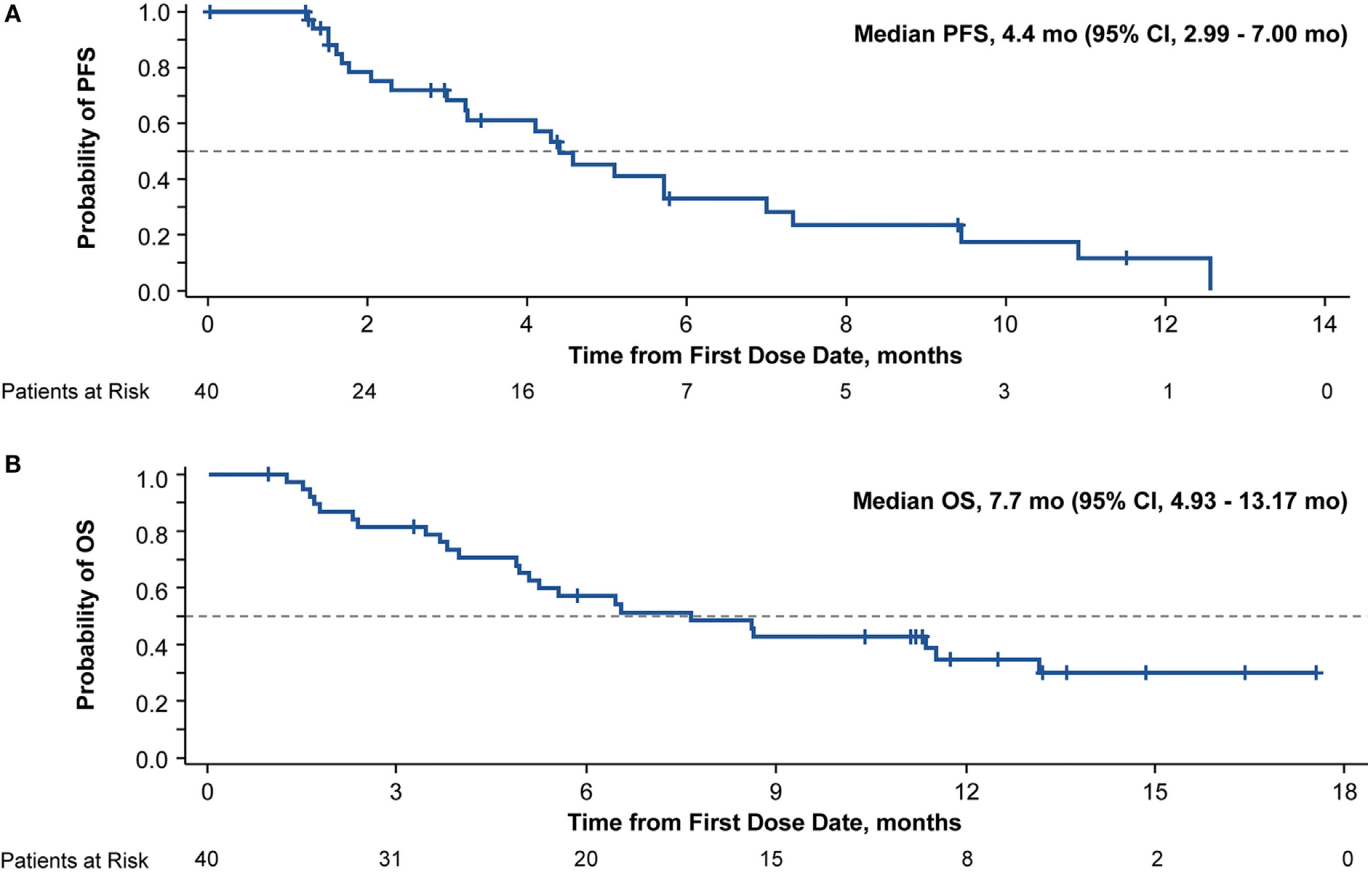
Kaplan–Meier curve of progression-free survival (PFS) **(A)** and overall survival (OS) **(B)** in patients with advanced non-small cell lung cancer and an Eastern Cooperative Oncology performance status of 2 treated with *nab*-paclitaxel-based therapy.

In all treated patients, 24 died or had disease progression. Median PFS was 4.4 months (95% CI, 2.99–7.00), and median OS was 7.7 months (95% CI, 4.93–13.17; Figure 1). The 6-month PFS rate was 33% (95% CI, 16–51%), and the 6-month OS rate was 57% (95% CI, 40–71%). The ORR was 30.0% (95% CI, 16.56–46.53), and the DCR was 75.0% (95% CI, 58.80–87.31); partial responses were observed in 12 patients, and 18 patients achieved stable disease. Of the 12 patients with a response, the median TTR was 2.0 months, and the median DOR was 6.8 months. Progressive disease as best overall response was observed in two patients. Among patients with a tumor assessment during induction (*n* = 32), 25.0% had partial responses; among patients with a tumor assessment at the end of cycle 2 (*n* = 31) and cycle 4 (*n* = 23), 19.4 and 30.4% had partial responses, respectively. Of the patients with a tumor assessment at the end of cycle 6 (*n* = 15), 40.0% had partial responses. Among the 16 patients entering monotherapy, 4 improved from stable disease to partial response; the remaining 12 patients had stable disease.

**Figure 2:**
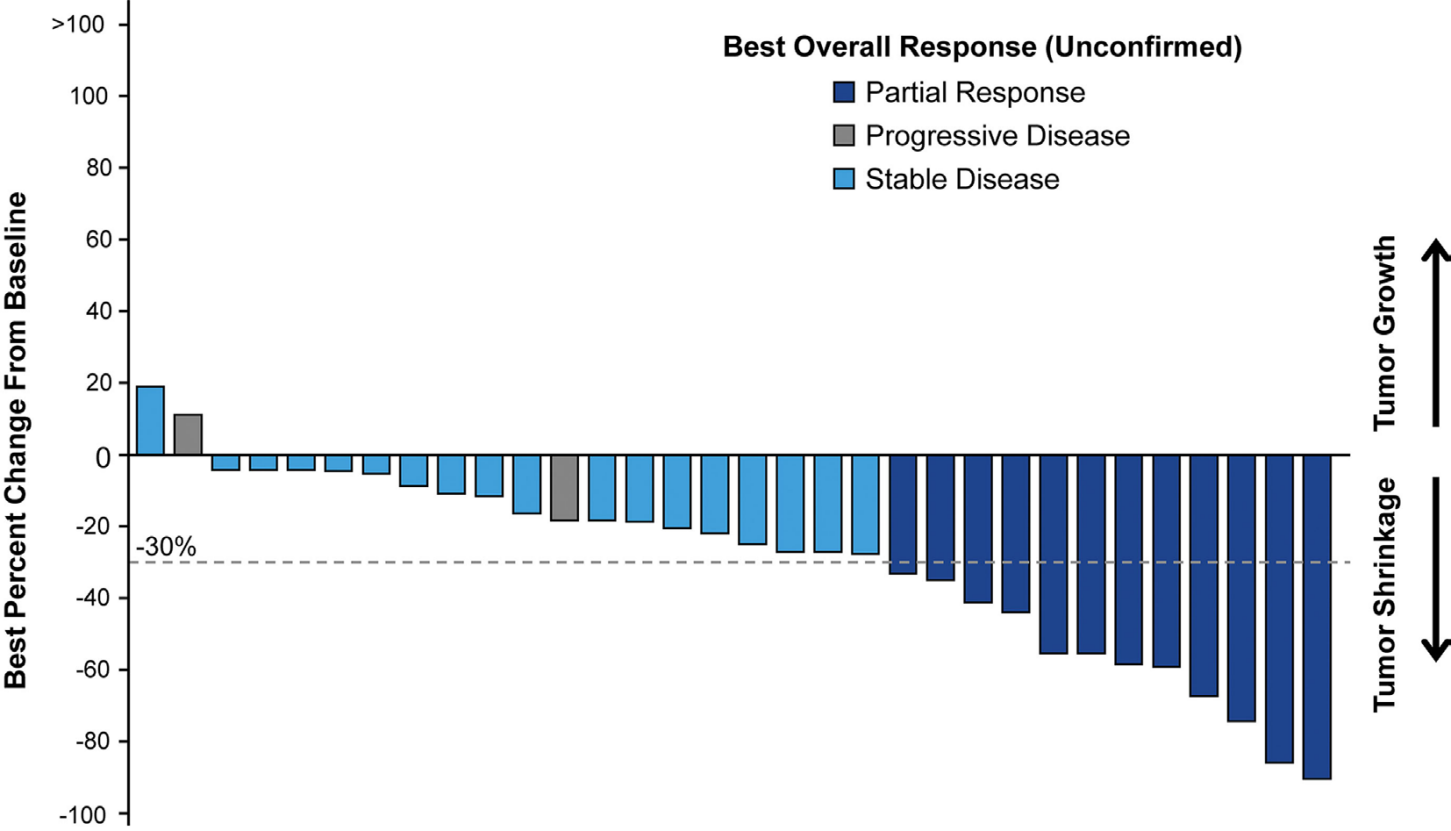
Waterfall plot of best percentage change from baseline in sum of diameter in target lesion with best overall response to *nab*-paclitaxel-based therapy in patient postbaseline tumor assessments.

Of patients with postbaseline tumor assessments (*n* = 32), the majority had tumor shrinkage; a best percentage target lesion decrease of ≥30.0% was observed in 12 patients (30.0%). The median best percentage change from baseline in the sum of diameters of target lesions was −22.6% (Figure 2).

### QOL and PS Status

In total, 34 patients completed baseline and ≥1 postbaseline QOL assessment. In general, symptom burden decreased, and a trend toward QOL improvement was observed during treatment. In the LCSS global QOL item scores, positive mean changes from baseline were observed at cycles 2–6, with clinically meaningful changes (mean ≥ 10) observed at cycles 3–6 (Figure S1A in Supplementary Material). The mean maximum improvement from baseline (at any point during treatment) in LCSS global QOL scores was 16.91 mm. The mean maximum improvement from baseline (at any point during treatment) in LCSS lung cancer symptoms was 7.21 points (SD, 25.06). Positive mean changes from baseline in LCSS normal activity scores were also observed at cycles 2–6 (Figure S1B in Supplementary Material). The mean maximum improvement from baseline (at any point during treatment) in the EQ-5D-5L VAS score was a positive mean change of 8.2 points (SD, 24.24). EQ-5D-5L dimensions remained stable or improved in the majority of patients (78.8–97.0%), with ≥30.0% of patients reporting complete resolution of problems reported at baseline at least once during treatment (Figure S2 in Supplementary Material). In addition, over the entire study, 62.5% of physician-reported ECOG PS remained the same, 25.0% improved by ≥1 level, and 5.0% improved by ≥2 levels; these values for patient-reported ECOG PS were 30.0, 27.5, and 7.5%, respectively.

### Longitudinal PS Assessment and Patient/Physician PS Concordance

Baseline ECOG PS score was reported as 2 by 47.5% of patients and 95.0% of physicians. At baseline, physicians believed that ECOG PS would be reversible with treatment in 80.0% of patients. Of the 38 patients with physician-assessed ECOG PS 2 at baseline, 9 (24%) improved to ECOG PS 1, and 2 (5%) improved to ECOG PS 0 at least once postbaseline. On cycle 1 day 1, only 52.8% of patients rated their ECOG PS score the same as did the physicians. For those with both pre- and posttreatment ECOG assessments, rates of patient-reported and physician-reported improvements from baseline at least once during treatment were 14 of 33 (42.4%) and 12 of 38 (31.6%), respectively.

### Lung Function

A total of 34 patients had baseline spirometry measurements (FEV_1_, FVC, FEV_1_/FVC ratio, PEF). In general, spirometry results indicated that pulmonary function remained stable over the course of treatment. The mean FEV_1_/FVC ratio remained stable over six cycles of treatment (Figure S3 in Supplementary Material), and a majority of patients had an improvement in mean PEF from baseline at least once during treatment (Figure S4 in Supplementary Material).

## Discussion

*nab*-Paclitaxel in combination with carboplatin as first-line treatment of locally advanced or metastatic NSCLC in patients with ECOG PS 2 was generally well tolerated, with favorable efficacy outcomes. Only ≈25% of patients discontinued treatment during induction due to TEAEs (primary endpoint), and efficacy—as measured by survival and response—was comparable to that seen in historical studies of chemotherapy in this patient population. The TEAEs reported during induction and monotherapy were consistent with the known safety profile of this chemotherapy combination and *nab*-paclitaxel alone. Among patients entering the monotherapy part, 25.0% improved from stable disease to partial response; the remaining patients had stable disease. Furthermore, QOL was generally stable or improved in most patients throughout the study.

Although few studies have been conducted in this patient subset, the results indicate that platinum doublets have acceptable toxicity and can benefit patients with poor PS. In a phase II trial, paclitaxel plus carboplatin treatment resulted in a median PFS and OS of 3.5 and 9.7 months, respectively, and an ORR of 12% (19). In the phase III STELLAR 3 trial, patients treated with paclitaxel plus carboplatin had a median OS of 7.9 months and an ORR of 37% (PFS not reported) (12). In the ECOG 1599 trial, a dose-attenuated paclitaxel plus carboplatin regimen resulted in a median PFS and OS of 3.5 and 6.2 months, respectively, and an ORR of 14% (16). Grade ≥3 toxicities in these studies were mainly hematologic, and grade ≥3 neuropathy (sensory or peripheral) occurred in 4–10% of patients. More recent prospective studies of other platinum doublets have demonstrated median OS values ranging from 5.8 to 6.7 months, and grade ≥3 toxicities were also generally hematologic in nature (10, 13–15). In the study evaluating pemetrexed with or without carboplatin, the response rates were 10.3% with pemetrexed and 23.8% with the combination (*P* = 0.032). The median PFS was 2.8 and 5.8 months [hazard ratio (HR), 0.46; 95% CI, 0.35–0.63; *P* < 0.001], and the median OS was 5.3 and 9.3 months in the pemetrexed and combination arms, respectively (HR, 0.62; 95% CI, 0.46–0.83; *P* = 0.001) (10). Efficacy results from the ABOUND.PS2 study were comparable to those in the prior studies, and TEAEs in the entire study were also mainly hematologic in nature; only one patient experienced grade 3 peripheral neuropathy. Furthermore, while immunotherapy is evolving as an important treatment option for advanced NSCLC, this regimen remains an alternative for patients who do not meet the criteria for first-line immunotherapy treatment.

Patients with advanced NSCLC and a poor PS also have a high disease burden and frequent comorbidities. Therefore, treatment dose and schedule selection are critical to balance toxicities while optimizing therapeutic benefit. In the ABOUND.PS2 trial, patients were treated with the approved dose of *nab*-paclitaxel (100 mg/m^2^) but on a different schedule (days 1 and 8, as opposed to the standard schedule of days 1, 8, and 15). The dose of carboplatin (AUC 5) was chosen on the basis of the phase III trial of pemetrexed vs pemetrexed plus carboplatin in patients with NSCLC and ECOG PS 2 (10). Furthermore, treatment of patients with four cycles of *nab*-paclitaxel plus carboplatin followed by *nab*-paclitaxel monotherapy is in line with prior studies evaluating platinum-doublet treatment in patients with advanced NSCLC and a poor PS (10, 14). Although the treatment schedule in the ABOUND.PS 2 study is similar to that of other maintenance schedules for patients with advanced NSCLC and good PS (20), the effects of continued maintenance therapy in prolonging tumor response or stable disease in patients with advanced NSCLC and ECOG PS 2 had yet to be evaluated. A potential advantage to the *nab*-paclitaxel–based regimen over a pemetrexed-based regimen is that the former can be administered to all patients with NSCLC, irrespective of histology. Furthermore, the need for vitamin supplementation and corticosteroids after chemotherapy is obviated. The ABOUND.PS2 study provided the unique opportunity to demonstrate the feasibility and tolerability of continuing *nab*-paclitaxel as maintenance chemotherapy after four cycles of induction.

The management of treatment- and disease-related symptoms is important in patients with advanced NSCLC and ECOG PS 2. In a phase II trial, measures of lung cancer-associated symptoms indicated that 15% of patients reported worsening of chest pain and 0% reported worsening of hemoptysis with platinum-doublet chemotherapy (paclitaxel plus carboplatin) compared with 37 and 24%, respectively, with erlotinib (19). Few other studies of platinum doublets have reported QOL results specifically for this patient population; therefore, more data would help clinicians understand the impact of treatment on QOL in patients with poor PS (14, 15). In the ABOUND.PS2 study, a trend toward improvement from baseline in patient QOL was observed in the LCSS global QOL item and the LCSS item that assesses ability to carry out normal activities, which are clinically relevant considerations for patients with advanced NSCLC and poor PS. Results from the current study add to the limited body of knowledge regarding the impact of platinum-doublet chemotherapy on QOL outcomes in patients with ECOG PS 2 and demonstrate that this *nab*-paclitaxel–based regimen did not negatively affect QOL in these patients. Furthermore, treatment with this *nab*-paclitaxel regimen resulted in ≈25% of patients with improvement in ECOG PS by ≥1 level over the entire study by both patient and physician assessment.

With the advent of checkpoint inhibitors, there is an expected decline in use and interest in platinum-based chemotherapy in the first-line setting. Notably, with greater use of checkpoint inhibitors in the first line, as demonstrated in KEYNOTE-042 study (21), there will still be a need and utility for platinum-based chemotherapy after progression on checkpoint inhibitors.

It is important to note the study limitations. ABOUND.PS2 was a small, single-arm study and did not reach planned accrual. Enrollment was slower than expected. Enrollment can be challenging in trials of patients with poor PS, and the introduction of novel agents, including immunotherapies, into the NSCLC treatment landscape can further negatively affect patient accrual in trials of chemotherapy. It should be noted that the decision to halt enrollment early was not based on any safety or efficacy concerns; a protocol-specified interim review deemed that sufficient data had been collected to support the planned analysis of the primary endpoint. Furthermore, the subjective nature of assigning PS may have contributed to the difficulty of enrolling a sufficient number of patients with ECOG PS 2 (11, 12, 19).

The results from ABOUND.PS2 further demonstrate the tolerability of a *nab*-paclitaxel-based combination induction and monotherapy maintenance treatment in patients with advanced NSCLC and ECOG PS 2. Although some oncologists may remain underwhelmed by these results and may choose to use pemetrexed and carboplatin in patients with nonsquamous histology, the current regimen offers a potential choice for patients with squamous histology and/or contraindications to pemetrexed. Efficacy outcomes were generally aligned with previous chemotherapy data, whereas improvements from baseline in several QOL measures were observed during treatment. In summary, this study provides support for the role of a *nab*-paclitaxel–based regimen in patients with NSCLC and ECOG PS 2.

*nab*^®^ is a registered trademark of Celgene Corporation.

## Data Availability Statement

Researchers may submit research proposals and/or request documents or anonymized data from Celgene clinical studies which support indications approved on or after 1 January 2014 in both the United States and Europe. Requests for information about other studies may also be accommodated and will be considered on a case-by-case basis based on availability.

## Abound.PS2 Investigators

**Ajeet Gajra**, SUNY Upstate Medical University, Syracuse, NY, United States. **David Robert Spigel**, Tennessee Oncology, PLLC, Nashville, TN, United States. **Deborah Mulford and Eric Kim**, University of Rochester Medical Center, Rochester, NY, United States. **Marc R. Matrana**, Ochsner Clinic Foundation, New Orleans, LA, United States. **Nagla Abdel Karim**, 200 Albert Sabin Way, Cincinnati, OH, United States. **Thomas M. J. Niederman**, University Cancer Institute, Boynton Beach, FL, United States. **Timothy Burns, Liza Villaruz**, and **Mark A. Socinski**, Hillman Cancer Center, Pittsburgh, PA, United States.

## Ethics Statement

This study was carried out in accordance with the recommendations of the institutional review board. The protocol was approved by the institutional review board. All subjects gave written informed consent in accordance with the Declaration of Helsinki.

## Author Contributions

All authors contributed to the conception and design of the work; contributed to the acquisition, analysis, or interpretation of data for the work; revised the manuscript critically for important intellectual content; approved the final version to be published; and agreed to be accountable for all aspects of the work in ensuring that all questions related to the accuracy or integrity of any part of the work are appropriately investigated and resolved.

## Conflict of Interest Statement

AG has been a consultant/advisor for Celgene, AstraZeneca, and Bayer. NK has served as a speaker for Novartis, Pfizer, and Prometheus and as an advisory board member for Bayer. MM received grant/research support from Astellas, Celgene, Genentech/Roche, Eli Lilly, and Pfizer; served as a consultant for Pfizer, Bayer, and EMD Serono; and served on a speaker’s bureau for Bristol-Myers Squibb, Eisai, Genentech/Roche, Merck, and Sirtex. ES served on a speaker’s bureau for AstraZeneca, Amgen, Boehringer Ingelheim, Celgene, Eli Lilly, Genentech, Roche, Merck, Millennium, Novartis, Pfizer, and Takeda. DS has been a consultant/advisor for Celgene, Genentech/Roche, Novartis, Bristol-Myers Squibb, Eli Lilly, AstraZeneca, Pfizer, Clovis Oncology, and Boehringer Ingelheim, and has received research funding from Genentech/Roche. TB, TO, AS, and KA are/were employed by/served in a leadership position and owned stock for Celgene. DM, LV, and HA have nothing to disclose.

## Abbreviations

AUC, area under the curve; CTCAE v4.0, Common Terminology Criteria for Adverse Events version 4.0; DCR, disease control rate; DOR, duration of response; ECOG, Eastern Cooperative Oncology Group; EQ-5D-5L, EuroQol 5 Dimensions 5 Levels; FEV_1_, forced expiratory volume in 1 s; FVC, forced vital capacity; HR, hazard ratio; LCSS, Lung Cancer Symptom Scale; NSCLC, non-small cell lung cancer; ORR, overall response rate; OS, overall survival; PEF, peak expiratory flow; PFS, progression-free survival; PS, performance status; QOL, quality of life; RECIST, Response Evaluation Criteria in Solid Tumors; TEAE, treatment-emergent adverse event; TTR, time to response; VAS, visual analog scale.
