# Supplementary figures and images for: nab-Paclitaxel–Based Therapy in Underserved Patient Populations: The ABOUND.PS2 Study in Patients With NSCLC and a Performance Status of 2
Source: Front Oncol. 2018 Jul 24;8:253. doi: 10.3389/fonc.2018.00253 (PMC6066533; doi:10.3389/fonc.2018.00253)

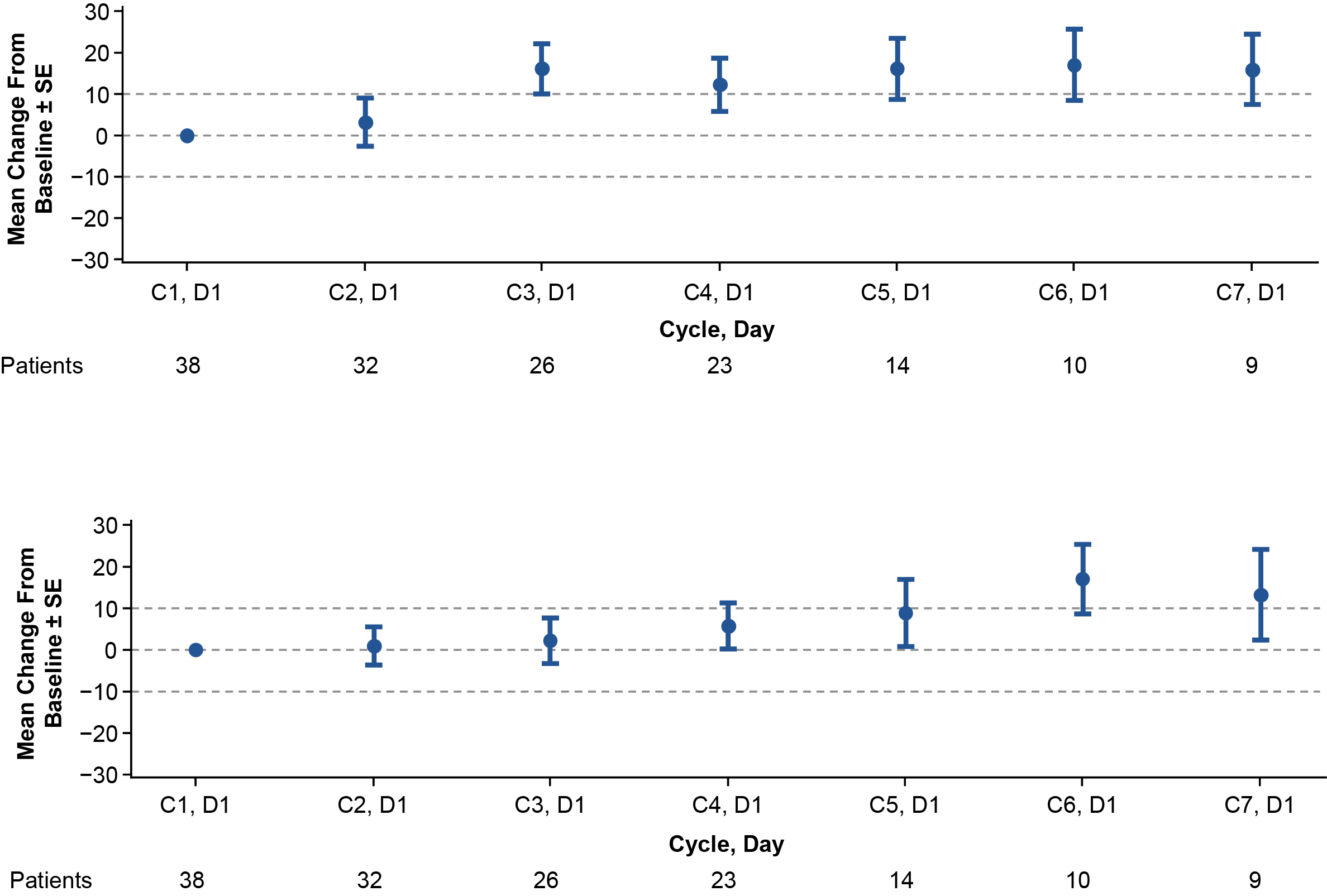

Supplement: Supplementary file 1 [file image_1.jpg]

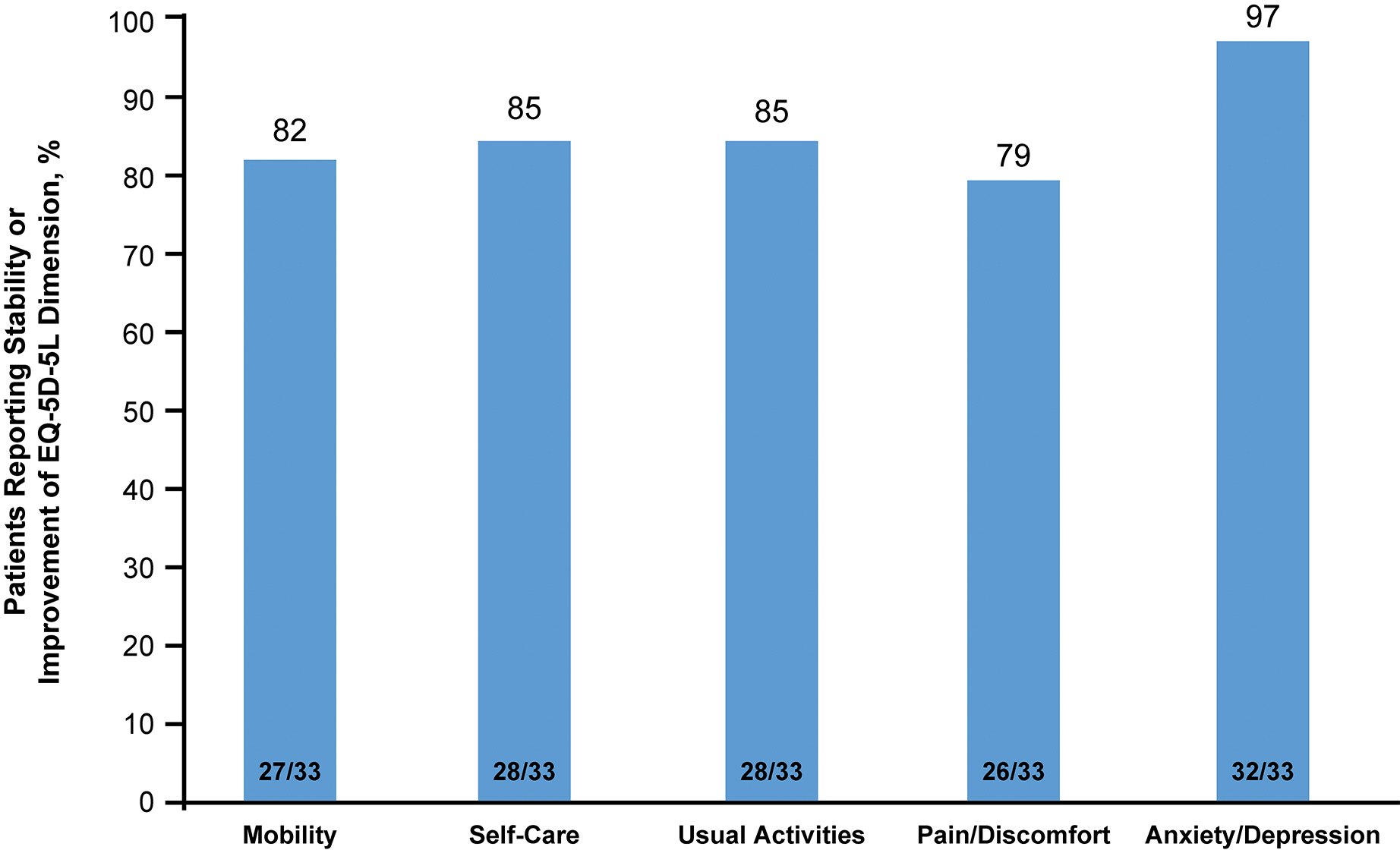

Supplement: Supplementary file 2 [file image_2.jpeg]

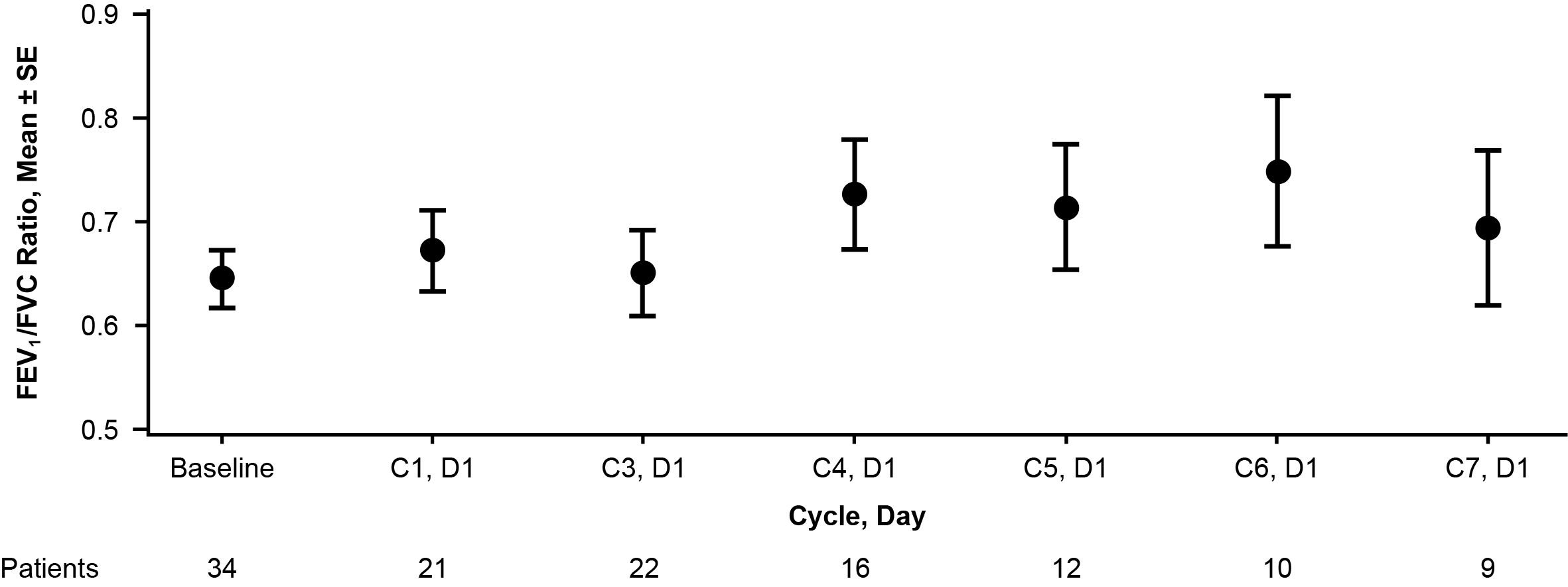

Supplement: Supplementary file 3 [file image_3.jpg]

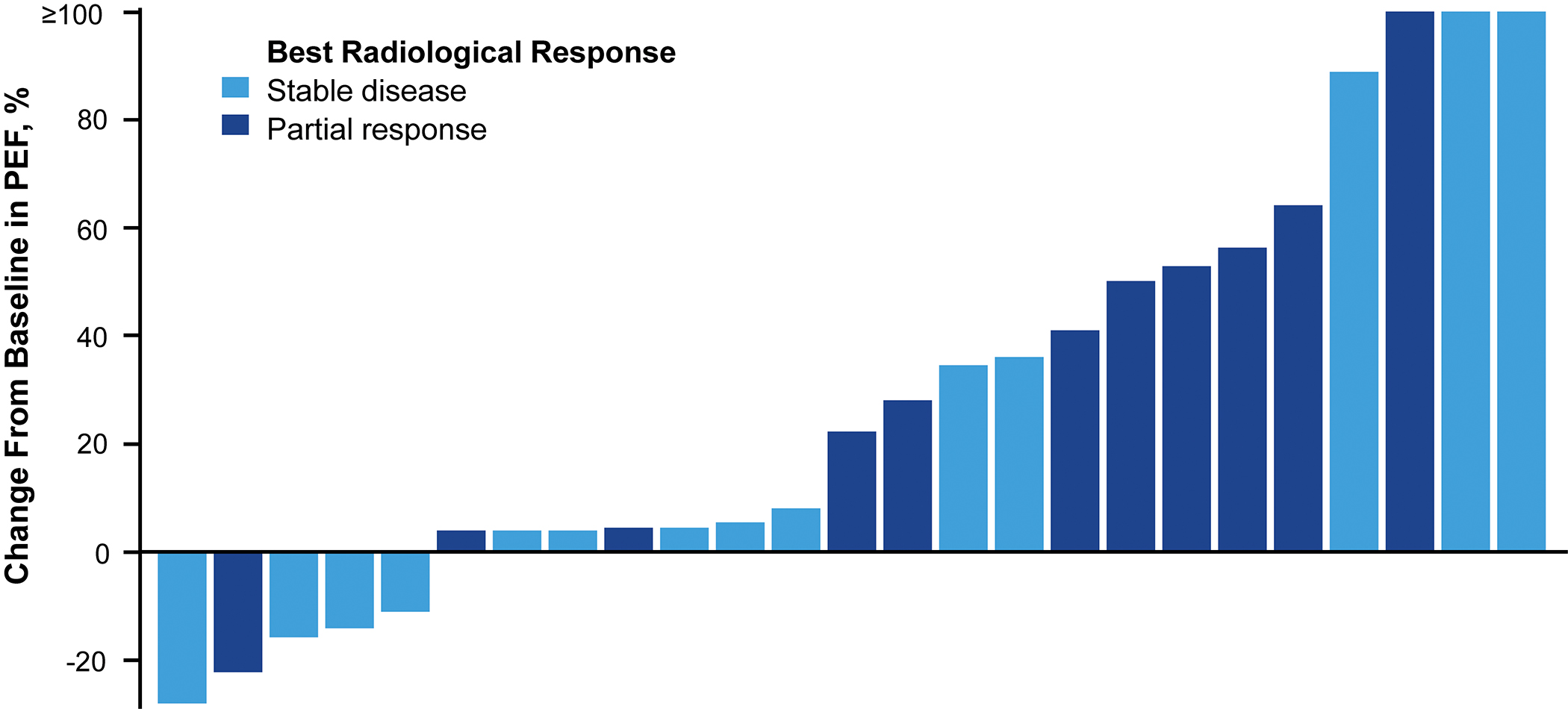

Supplement: Supplementary file 4 [file image_4.jpeg]
